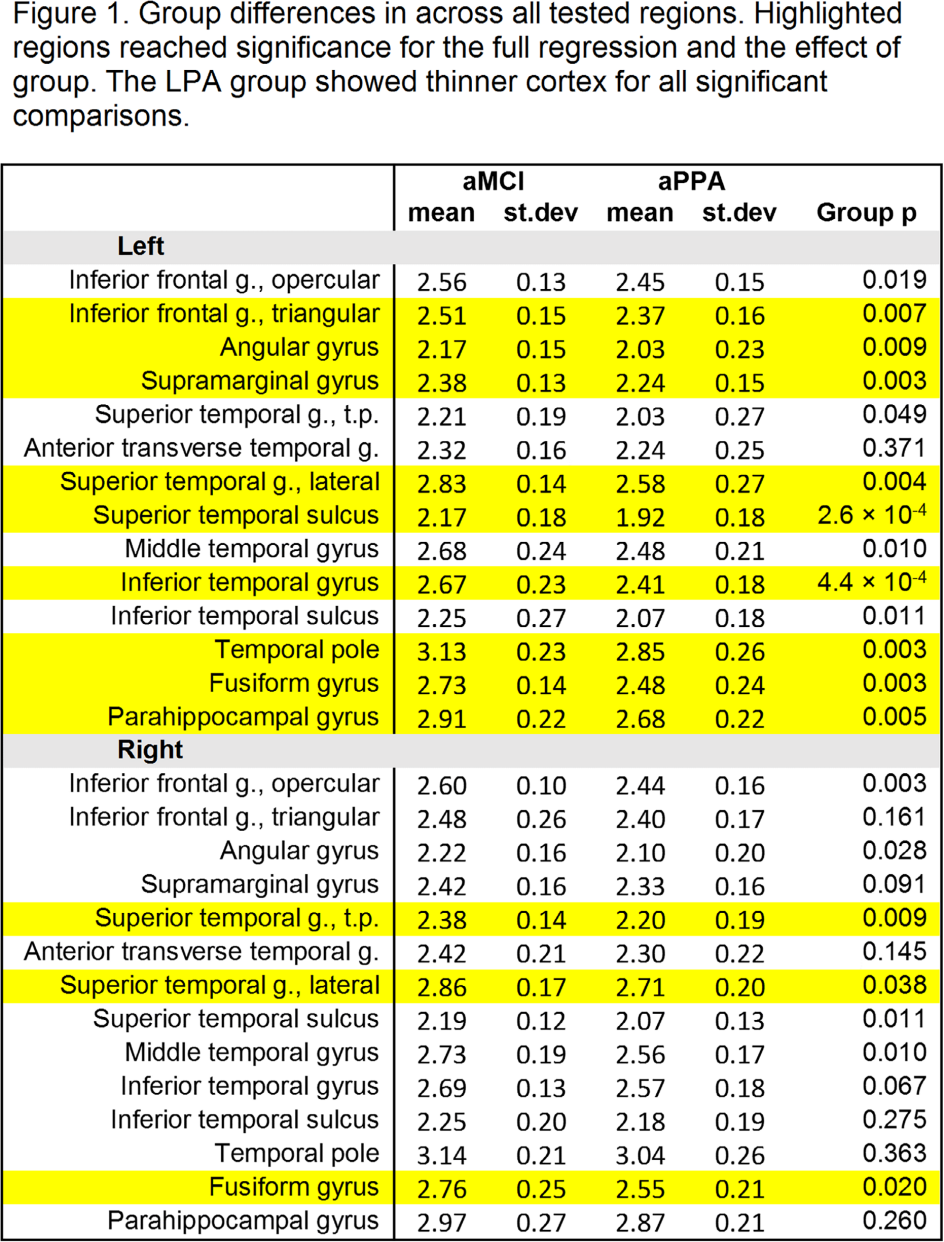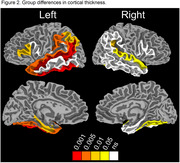# Regional cortical thinning in amnestic MCI and logopenic primary progressive aphasia for similar symptom duration

**DOI:** 10.1002/alz70856_103275

**Published:** 2025-12-26

**Authors:** Katherine A Koenig, Xuemei Huang, Aaron Bonner‐Jackson, James B Leverenz, Mark J Lowe, Jagan A. Pillai

**Affiliations:** ^1^ Cleveland Clinic, Cleveland, OH, USA; ^2^ Cleveland Clinic Neurological Institute, Cleveland, OH, USA; ^3^ Cleveland Clinic Lou Ruvo Center for Brain Health, Cleveland, OH, USA; ^4^ Cleveland Clinic Lou Ruvo Center for Brain Health, Las Vegas, NV, USA

## Abstract

**Background:**

Logopenic primary Progressive Aphasia (LPA) is characterized by early language‐related decline in neurodegenerative conditions and is often seen in Alzheimer's disease (AD). LPA has been described to have predominant left posterior tempero‐parietal atrophy compared to other types of primary progressive aphasias. Here we determine MRI cortical thickness (CT) changes in LPA from AD that are distinct and those that overlap with amnestic MCI from AD (aMCI) and related neuropsychology (NP) correlates.

**Method:**

Thirteen aMCI (mean age 67.5 ± 5.9; 6 males) and twenty‐six LPA patients (mean age 68.0 ± 7.93 years; 13 males) were scanned on a Siemens 3T MRI scanners. The anatomical scan was segmented using Freesurfer 7.1.4. For each participant, measures of interest included regional CT as detailed in Figure 1. Regressions correcting for age, sex, and education were used to calculate group differences. Measures in regions that showed a significant group difference were correlated with neuropsychological (NP) measures within each group.

**Result:**

The groups were matched on age (*p* < 0.838), education (*p* < 0.345), sex (0.821), and symptom duration (0.617). Figure 1 shows all tested regions, with highlighted regions significant for the full regression and the effect of group. The LPA group had thinner cortex than the aMCI group in all significant regions, with most differences seen in the left hemisphere (Figure 2) but also included temporal and fusiform areas in the right. In the aMCI group, thickness of the left superior temporal gyrus was related to performance on recall, learning, auditory attention, and executive function measures (*p* < 0.05). Significant correlations to NP measures were more distributed in the LPA group, with significant regions including the left fusiform, parahippocampal, and angular gyri in addition to the superior temporal gyrus.

**Conclusion:**

For a similar symptom duration, we found significantly thinner cortex in language and AD‐related regions in LPA compared to aMCI, predominately in the left hemisphere. Correlations to NP measures were more widely distributed in the LPA group than aMCI. An understanding of brain changes specific to LPA and aMCI will allow better characterization of the neurodegenerative process in relation to rate of disease progression.